# Aggressive Natural Killer Cell Leukemia: A Rare and Rapidly Progressive Hematologic Malignancy—Case Report and Literature Review

**DOI:** 10.1155/crh/7796972

**Published:** 2026-04-06

**Authors:** Jennifer Priessnitz, Ali Hariri, Yuliya Levkiavska, Kyle E. Bonner, Stephen I. Fisher, Joshua M. Sill

**Affiliations:** ^1^ Department of Internal Medicine, Eastern Virginia Medical School at Old Dominion University, Norfolk, Virginia, USA, evms.edu; ^2^ Department of Medicine, Eastern Virginia Medical School at Old Dominion University, Norfolk, Virginia, USA, evms.edu; ^3^ Department of Internal Medicine, Division of Pulmonary Critical Care Medicine, Eastern Virginia Medical School at Old Dominion University, Norfolk, Virginia, USA, evms.edu; ^4^ Department of Diagnostic Radiology, Eastern Virginia Medical School at Old Dominion University, Norfolk, Virginia, USA, evms.edu; ^5^ Department of Pathology, Division of Hematopathology, Pathology Sciences Medical Group, Sentara Norfolk General Hospital, Norfolk, Virginia, USA

**Keywords:** aggressive natural killer cell leukemia, case report, Epstein–Barr virus, hemophagocytic lymphohistiocytosis, targeted therapy

## Abstract

Aggressive natural killer cell leukemia (ANKL) is a rare, fulminant hematologic malignancy characterized by neoplastic proliferation of mature NK cells. It is frequently associated with Epstein–Barr virus (EBV) infection, although EBV‐negative cases have also been reported. While typically observed in young to middle‐aged adults of East Asian descent, increasing recognition has led to identification of ANKL across diverse age groups and ethnicities. The disease is defined by a rapid clinical course and poor prognosis, underscoring the importance of early diagnosis and effective treatment. We present a case of a 71‐year‐old Caucasian male who developed fever, altered mental status, hepatosplenomegaly, pancytopenia, and hemophagocytic lymphohistiocytosis (HLH) and who was ultimately diagnosed with ANKL. Although he demonstrated initial clinical improvement with chemotherapy, relapse of the ANKL ensued a few months later, and he ultimately succumbed to progressive disease. A review of current literature is provided, focusing on molecular pathogenesis and emerging therapeutic strategies.

## 1. Introduction

Aggressive natural killer cell leukemia (ANKL) is a rare and highly lethal hematologic malignancy characterized by systemic proliferation of mature NK cells, most frequently associated with Epstein–Barr virus (EBV) infection. Due to its rapidly progressive course, median survival is typically only a few weeks to months, and early relapse is nearly universal, even after intensive chemotherapy [1, 2]. Hematopoietic stem cell transplantation (HSCT) remains the only potentially curative option, although its feasibility is often limited by early clinical deterioration [1, 2].

In the WHO 5th edition classification, ANKL was defined as a systemic lymphoid neoplasm characterized by NK‐cell immunophenotype and absence of T‐cell receptor protein expression and/or clonal TCR rearrangement [3, 4]. Although it represents fewer than 1% of all non‐Hodgkin lymphomas and has fewer than 200 cases reported in the literature, recognition is increasing [5]. While historically most prevalent in East Asian populations, cases have been documented across other racial and ethnic groups, including Caucasian and Latin American individuals, and across a wide age spectrum from children to older adults [2, 6, 7]. Common clinical features include fever, cytopenias, hepatosplenomegaly, and constitutional B symptoms. ANKL frequently presents with or evolves into hemophagocytic lymphohistiocytosis (HLH), which is a hyperinflammatory syndrome resulting from excessive cytotoxic lymphocyte and macrophage activation, mediated through perforin and granzyme release, leading to multiorgan dysfunction [8, 9].

The diagnosis of ANKL remains challenging due to overlap with other hematologic and infectious conditions. The hallmark diagnostic findings include NK‐cell phenotype (ccluster of differentiation 2 (CD2^+^), CD56^+^, surface CD3^−^) with expression of cytotoxic granule proteins such as TIA‐1 (T‐cell intracellular antigen‐1), granzyme B, and perforin [10]. In 90% of all ANKL cases, EBV is typically detected via EBV‐encoded RNA (EBER) in situ hybridization (ISH) or EBV RNA by targeted RNA next‐generation sequencing (EBV tRNA NGS) [11].

In the context of HLH, supportive serologic findings include hyperferritinemia, hypertriglyceridemia, and elevated soluble interleukin (IL)‐2 receptor (sCD25) and CXCL9 levels. Genomic studies have identified frequent mutations in STAT3, TP53, TET2, and epigenetic regulators, offering insights into disease biology and potential targets for therapy [10, 12].

We present a case of ANKL in a 71‐year‐old Caucasian male with HLH as the initial manifestation, highlighting diagnostic complexities in an atypical demographic. We also provide a comprehensive literature review emphasizing evolving molecular insights and investigational treatment strategies.

## 2. Case Presentation

**FIGURE 1:**
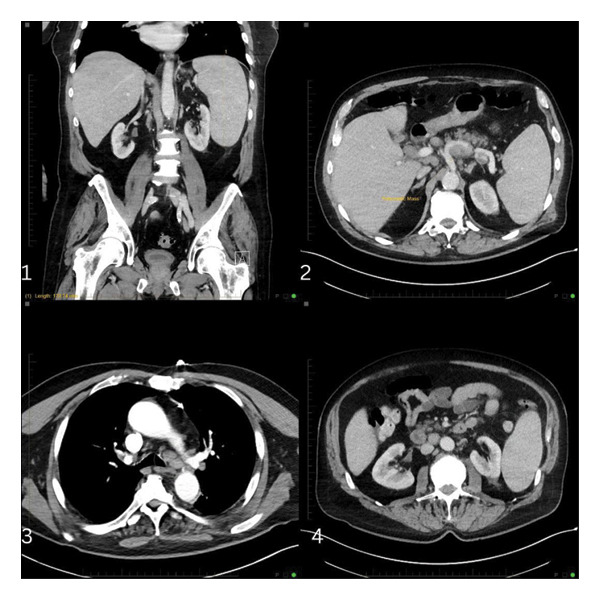
CT chest, abdomen, and pelvis with contrast at presentation revealed (image 1) splenomegaly measuring up to 17 cm in craniocaudal dimension, (image 2) a 4 cm nodular density within the pancreatic tail abutting the left splenic artery, (image 3) extensive lymphadenopathy including bilateral hilar and mediastinal nodes, and (image 4) additional lymph nodes in the porta hepatis measuring up to 21 mm.

A 71‐year‐old Caucasian male with a history of coronary artery disease, status postcoronary artery bypass graft, and aortic valve replacement presented with several months of progressive dyspnea, fatigue, confusion, visual hallucinations, and cyclical fevers. Formerly a practicing physician, he experienced a precipitous decline in mental status over the past week. Abdominal computed tomography (CT) revealed splenomegaly, a pancreatic mass, and extensive retroperitoneal and hilar lymphadenopathy (Figure 1). Head CT was unremarkable. Laboratory findings were notable for anemia (hemoglobin 6.8 g/dL), thrombocytopenia (platelet count 38 K/μL), acute kidney injury (creatinine 1.6 mg/dL), and markedly elevated ferritin (3339 ng/mL).

He was empirically treated for presumed sepsis with broad‐spectrum antibiotics but clinically deteriorated by hospital day three, developing hypoxic respiratory failure, hypotension, lactic acidosis (lactate 5.3 mmol/L), and oliguric renal failure necessitating hemodialysis. Workup for infectious and neurologic etiologies was unrevealing (e.g., blood cultures, lumbar puncture, magnetic resonance imaging [MRI] of the brain, and peripheral smear); however, given the constellation of cytopenias, hyperferritinemia, hypertriglyceridemia, and detectable EBV (EBV PCR Quant positive at 729 copies/mL), HLH was suspected. Serologies for soluble CD25 and CXCL9 were sent. Due to significant pancytopenia, clinical instability, and the high mortality rate of HLH, the decision was made to initiate high‐dose steroids and etoposide, while awaiting bone marrow and lymph node biopsies. Additional laboratory findings included elevated neuron‐specific enolase (140 ng/mL), chromogranin A (409 ng/mL), and procalcitonin (3.4 ng/mL). Serum serotonin was low (13 ng/mL) and A disintegrin and metalloproteinase with thrombospondin motifs 13 (ADAMTS13) activity was mildly reduced (0.37 IU/mL). Serum‐free lambda light chains were elevated (30.2 mg/L), with a kappa‐to‐lambda ratio of 0.64; immunoglobulin G (IgG) level was 640 mg/dL. Doxycycline and rifampin were discontinued as atypical infectious causes were thought to be unlikely.

**FIGURE 2:**
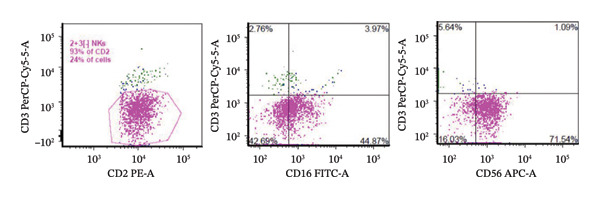
Retroperitoneal lymph node flow showed 24% atypical T‐cells expressing CD2+, CD16[dim]+, and CD56+. Negative for CD3−, [CD4, CD5, CD7, CD8, and CD45; not shown].

**FIGURE 3:**
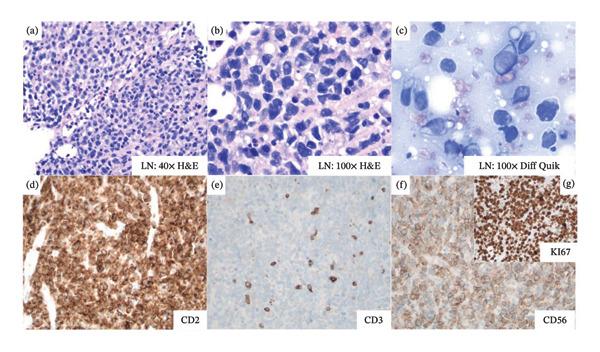
Lymph node stained with hematoxylin and eosin at 40x magnification (a) and 100x magnification (b). Lymph node smear stained with “diff quick” at 100x magnification revealed moderate amounts of pale‐basophilic, agranular cytoplasm with few small, punched out cytoplasmic vacuoles, ovoid to irregular nuclear contours, and a mature chromatin pattern with few nucleoli (c). Lymph node showed sheets of large lymphoid cells positive for CD2+ (d), CD3[ε]+ (e), and CD56+ (f) by immunohistochemistry staining. KI67+ expression was very high (100%) (g).

On hospital day nine, following clinical stabilization, the patient underwent image‐guided retroperitoneal lymph node and bone marrow biopsies. Retroperitoneal lymph node flow cytometry showed 24% atypical T cells expressing CD2+, CD16[dim]+, and CD56+. Negative for CD3− (Figure 2). Additionally, the lymph node biopsy was positive for CD56+, and the KI67 proliferative index was high [100%] by immunostaining (Figure 3).

**FIGURE 4:**
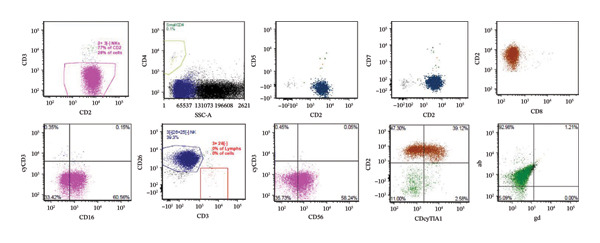
Peripheral blood flow cytometry revealed 28% atypical NK cells expressing CD2+, CD16+, CD26+, CD56+, cyTIA1+ [CD38, CD45, HLADR; not shown]; negative for CD3−, cyCD3−, CD4−, CD5−, CD7−, CD8−, TCR γδ−, and TCR αβ− [CD11b, CD25, p53, and CD57; not shown].

**FIGURE 5:**
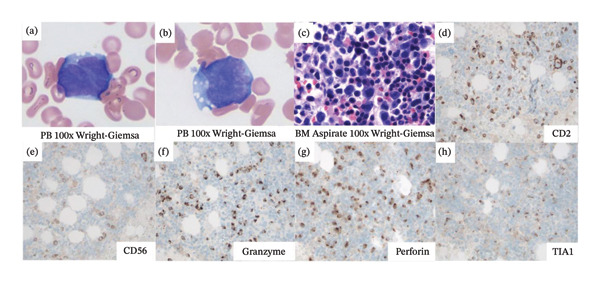
Peripheral blood smear Wright‐Giemsa stain at 100x magnification showed a population of large lymphoid cells with convoluted nuclei, mature chromatin, absent nucleoli, and cytoplasmic vacuoles (a, b). Bone marrow aspirate Wright‐Giemsa stain showed scattered large, irregular lymphoid cells with occasional nucleoli and scant to moderate cytoplasm (c). Bone marrow clot sections showed scattered medium to large, irregular lymphoid cells positive for CD2+ (d), CD56+ (e), and cytotoxic markers granzyme B (f), perforin (g), and TIA‐1 (h), by immunohistochemistry (negative for CD3− and CD7−; not shown).

Peripheral blood flow cytometry revealed 32% atypical NK cells expressing CD2+, CD16+, CD26+, CD30 [partial]+, CD38+, CD45+, CD56+, HLADR+, cyTIA1+, CD3−, cyCD3−, CD4−, CD5−, CD7−, CD8−, CD11b−, CD25−, p53−, CD57−, TCR αβ−, TCR γδ− immunophenotype (Figure 4), and peripheral blood smear review showed a large abnormal lymphoid cell population (Figure 5).

**FIGURE 6:**
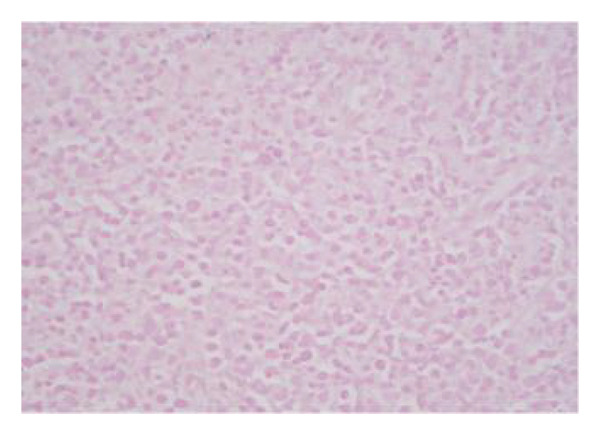
Lymph node with negative staining for EBER ISH was negative. This was thought to be due to the effects of high dose steroids.

Additionally, the bone marrow aspirate demonstrated a hypercellular marrow with erythroid hyperplasia and 28% atypical lymphoid cells; overt hemophagocytosis was not observed (Figure 5). The bone marrow sections showed large, irregular lymphoid cells with occasional nucleoli and scant to moderate cytoplasm positive for CD2+, CD56+, CD3−, CD7−, and by immunohistochemistry (IHC) they were positive for P53 and cytotoxic markers granzyme B, perforin, and TIA‐1 (Figure 5). EBER ISH was negative for EBV in the lymph node biopsy (Figure 6). Of note, it is theorized that negative EBER ISH staining and absence of hemophagocytosis in bone marrow biopsy were due to the effect of high‐dose dexamethasone and etoposide administered for HLH prior to biopsy.

Bone marrow FISH studies showed a complex karyotype with the following chromosomal abnormalities: 3 copies of 5 without deletion of 5q; 3 to 5 copies of 7 without deletion of 7q; 3 copies of 8; 3 copies of 13 without deletion of 13q; no deletion of p53 (17p13) and 3 to 4 copies of 20q. Bone marrow cytogenetic studies revealed a highly complex hyperdiploid karyotype with the following chromosomal aberrations: (74 ∼ 76< 3n >, XXYY, add (X) (p11.2), der(1; 3) (p10; p10), add (1) (q21), add (5) (q35), add (6) (p23), +7, add(8) (p21),+9, add (9) (p12), del (11) (q13q23),+12,+14,−17,−18,+20,+21,+22,+2mar [cp7]/46, XY [13]). Molecular NGS studies identified mutations in SUFU, TP53, CREBBP, FAS (2 mutations), TET2 (2 mutations), ARID2, BCOR, SMAD2, SRSF2, and RECQL4 genes, t(3; 17) (p11; p12) NCOR1:CHMP2B fusion mRNA, t(8; 17) (p23; p13) TNKS::GAS7 fusion mRNA. Autosomal chromosomal structural analysis shows numerous abnormalities including small 6q−, +7, +9, +12, small 17p+, −18. B and T cell clonality not detected. Increased CD2, CD3Z, CD16, CD56, GZMB, PRF1, KI67 mRNA. Low‐level CD3D, CD3G, CD4, CD5, CD7, CD8, CD30, and CD57 mRNA.

The above immunophenotypic, FISH, cytogenetic, and genomic studies support the diagnosis of ANKL.

**FIGURE 7:**
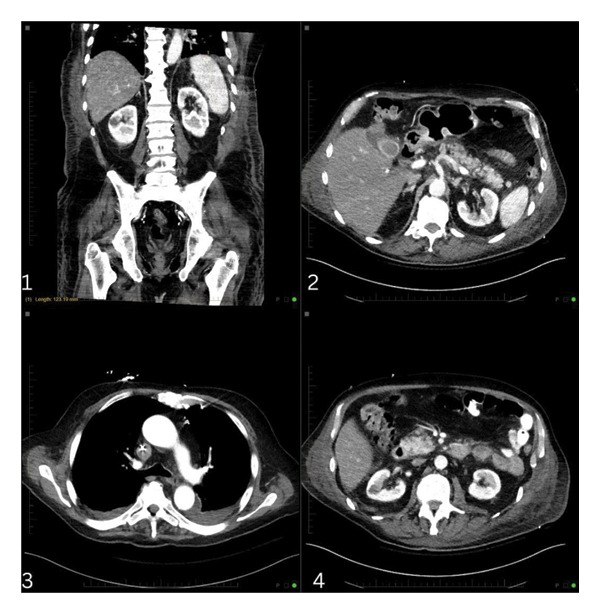
CT chest, abdomen, and pelvis with contrast: showed (image 1) resolution of splenomegaly (now 12.3 cm), (image 2) complete resolution of 4 cm nodular density within the pancreatic tail, (image 3) resolution of extensive lymphadenopathy, and (image 4) resolution of periaortic lymphadenopathy.

On hospital Day 12, chemotherapy was initiated with a renally dosed etoposide, ifosfamide, dexamethasone, L‐asparaginase, VIDL‐based regimen consisting of etoposide, ifosfamide, mesna, dexamethasone, and later L‐asparaginase. Laboratory makers also confirmed the clinical suspicion of HLH: elevated soluble CD25 (86,625 pg/mL) and CXCL9 (120,173 pg/mL) (Table 1). Over the following two weeks, the patient showed significant clinical improvement with normalization of mental status, recovery of renal function, and successful extubation. Imaging showed resolution of splenomegaly, pancreatic mass, and lymphadenopathy (Figure 7). A repeat bone marrow biopsy on Day 37 showed no evidence of residual ANKL. The patient’s full diagnostic workup is shown in the Supporting Information.

**TABLE TABLE​ 1:** The patient’s HLH diagnostic criteria.

HLH 2004 diagnostic criteria [**9**]	
	**Our patient**

The diagnosis of HLH can be established if one of the either 1 or 2 below is fulfilled:	
1) A molecular diagnosis consistent with HLH	No
2) Diagnostic criteria for HLH fulfilled (5 of the 8 criteria below)	
Fever	Yes, 38.3 C
Splenomegaly	Yes
Cytopenias (≥ 2 of 3 lineages in the peripheral blood):	
Hemoglobin < 9.0 g/dL	Hb: Yes, 6.0 g/dL
Platelets < 100 K/uL	Plts: Yes, 24 K/uL
Neutrophils < 1.0 K/uL	Neutrophils: No, 4.5 K/uL
Hypertriglyceridemia and/or hypofibrinogenemia: fasting triglycerides ≥ 265 mg/dL, fibrinogen < 1.5 g/L	Triglyceride: Yes, 615 mg/dL Fibrinogen: No
Hemophagocytosis in bone marrow, spleen, liver, or lymph nodes	∗No (patient was on high dose dexamethasone for three days prior to bone marrow biopsy for presumed HLH, likely altering results)
Low or absent NK cell activity	Not measured
Ferritin > 500 ng/mL	Yes, 3339 ng/mL
Soluble CD25 ≥ 2400 pg/mL	Yes, 120,173 pg/mL
*Supportive evidence*: Central nervous system (CNS) symptoms, hepatitis, renal failure, respiratory failure	Yes, positive for CNS symptoms, renal failure, respiratory failure

Unfortunately, after only four weeks, his condition began to deteriorate. He developed worsening cytopenias and hepatic dysfunction. Repeat flow cytometry revealed reemergence of an aberrant NK‐cell population (2.5%). Despite supportive care, the patient’s condition rapidly worsened. Re‐induction chemotherapy and bone marrow transplant were considered; however, the decision was made to transition to comfort care measures. He passed away shortly after.

## 3. Discussion

### 3.1. Presentation

Patients with ANKL typically present with constitutional symptoms such as fever, fatigue, altered mental status, weight loss, and other constitutional B symptoms. Hepatomegaly and splenomegaly are also frequent findings [2, 10]. Other clinical features can include lymphadenopathy, extramedullary masses, anemia, and thrombocytopenia [10]. Laboratory findings often include markedly elevated lactate dehydrogenase, reflecting tumor burden. Although primarily a marrow‐based malignancy, extramedullary involvement can occur, including the skin, lungs, and central nervous system [7, 13]. Our patient’s cyclical fevers, hepatosplenomegaly, cytopenias, and multiorgan dysfunction were consistent with classical ANKL presentations.

### 3.2. Epidemiology

ANKL is an exceptionally rare malignancy, with fewer than 200 cases described in the literature, accounting for < 1% of all non‐Hodgkin lymphomas [2, 10]. While initially believed to predominantly affect East Asian populations, more recent data demonstrate increasing prevalence in Caucasian and Latin American patients [14]. ANKL typically affects young to middle‐aged adults (median age ∼40 years), though cases in children and older adults have been documented [15, 16]. Recent data from U.S.‐based cohorts reveal a broader demographic distribution, with a substantial proportion of cases occurring in Caucasians (76.4%) and older adults [6]. The disease appears to occur with equal gender distribution, although some sources suggest male predominance in certain populations [15].

### 3.3. Diagnosis

Diagnosis of ANKL requires integration of clinical, morphologic, immunophenotypic, and molecular data. The main differential diagnosis can include chronic lymphoproliferative disorder of NK cells, chronic active EBV, EBV‐positive T‐cell and NK‐cell lymphoproliferative diseases of childhood, ENKL, and rarely other EBV‐associated T‐cell lymphomas [3, 4]. In ANKL, bone marrow aspirates typically reveal large granular lymphocytes [10]. IHC studies show the characteristic NK‐cell phenotype: CD2+, sCD3−, CD3ε+, CD5−, CD7+, CD16+, CD56+, and positive for cytotoxic molecules (granzyme B, perforin, and/or TIA1). CD8 and CD11b may be expressed, whereas CD57 is usually negative. Notably, the absence of a TCR gene rearrangement by PCR and/or NGS is requisite [3, 4].

The presence of EBV infection by EBER ISH, EBV DNA by qPCR, or EBV RNA by NGS in the neoplastic cells suggests that EBV may contribute to the pathogenesis of ANKL and supports this diagnosis in EBV‐associated cases; however, the exact etiology and ANKL remains unknown [3, 4, 7]. Distinguishing ANKL from extranodal NK/T‐cell lymphoma, nasal type (ENKTL), is imperative due to differing therapeutic approaches and clinical behavior [17]. Unlike ENKTL, which is more localized to the nasal sinuses and cutaneous skin and radiation‐sensitive, ANKL exhibits fulminant systemic progression, requiring immediate cytotoxic therapy. Expression of CD16 is characteristic of ANKL contrary to ENKL, suggesting a distinct differentiation of stage of NK cells [18, 19]. The abnormal NK cell immunophenotype provides a key diagnostic basis for ANKL, and flow cytometric immunophenotyping is sensitive for the early diagnosis of de novo ANKL [2, 20].

### 3.4. Association With EBV

ANKL is strongly associated with EBV, with > 85%–90% of cases testing positive for EBER or EBV RNA [7, 11]. High EBV DNA (qPCR) and RNA (tRNA NGS) levels can also raise suspicion for ANKL, and some studies suggest that EBV‐positive ANKL patients are more likely to have multiorgan involvement and HLH [21, 22]. Additionally, monitoring EBV DNA levels in the blood can be useful clinically, as they correlate well with disease activity [22].

Interestingly, programmed cell death protein 1 (PD‐1) inhibition may represent a promising approach for ANKL treatment, and this may be particularly true in EBV‐positive cases as EBV infection plays a role in programmed death‐ligand 1 (PD‐L1) stimulation [10]. Notably, EBV‐negative cases, such as those described by Nicolae et al., may present with a slightly more indolent disease course [7]. In our case, the absence of EBER‐positive staining may indicate a partially EBV‐independent pathogenesis or more likely reflect treatment‐related suppression, complicating both classification and prognosis.

### 3.5. Molecular Pathogenesis and Biomarkers

The exact etiology of ANKL remains poorly understood, due in part to its extremely rare nature. EBV‐positive ANKL cells typically exhibit a clonal episomal form of EBV and overexpress IL‐10, contributing to JAK/STAT activation [23]. EBV is thought to evade the immune system through a Type I latency pattern of infection in which neoplastic NK cells are not detected by host virus‐specific cytotoxic T cell activity [1, 10]. The interplay between this heightened immunogenic leukemic state secondary to EBV proliferation and EBV’s high association with the development of HLH could explain why many ANKL patients present with or develop HLH [21].

Key molecular abnormalities include activation of the JAK/STAT pathway (e.g., STAT3 or STAT5B) resulting in cell proliferation and resistance to apoptosis and cytokine signaling; although distinguishing ANKL from ENKL, no JAK3 mutations have been discovered [24]. TP53 mutations lead to loss of p53 protein expression, disrupting DNA damage response and loss of cell cycle checkpoint control. Mutations in KRAS lead to RAS/MAPK signaling, which promotes oncogenic signaling and cell cycle progression. Mutations in ASXL1, ASXL2, and BRINP3 lead to impaired DNA damage repair, leading to genetic aberrations and malignant transformation. Mutations in TET2, CREBBP, and ARID2 reflect disruption of epigenetic regulation, affecting DNA methylation and chromatin remodeling which silences tumor suppressor genes or activates oncogenes. Mutations in PRPF40B lead to dysregulation of mRNA splicing, leading to lymphomagenesis by dysfunctional protein transcripts [10, 25]. Chromosomal aberrations in ANKL include gains in 1q23.1 and 1q23.2, as well as losses in 7p15 and 17p13. Frequent loss of p53 expression, overexpression of BCL‐2 (antiapoptotic) and MYC (transcriptional driver of proliferation and metabolism), and dysregulation of the JAK/STAT–MYC–biosynthesis axis have been shown in ANKL cases [10, 25, 26].

### 3.6. Treatment of ANKL

ANKL is characterized by an extremely poor overall survival, and due to the rarity and aggressive nature of ANKL, there is no established, standardized treatment. L‐asparaginase‐based combination chemotherapy regimens, often followed by allogeneic HSCT, appear to improve overall survival and are considered the only potential curative option [1]. The efficacy of L‐asparaginase in chemotherapeutic regimens and its association with NK‐cell apoptosis have led to its inclusion in standard regimens such as L‐asparaginase, methotrexate, and dexamethasone; etoposide, ifosfamide, dexamethasone, and L‐asparaginase; or dexamethasone, ifosfamide, cisplatin, and etoposide [1]. Concurrent chemoradiation with VIDL has shown excellent clinical response. Sequential chemoradiation with steroids, methotrexate, ifosfamide, L‐asparaginase, and etoposide (SMILE) has shown an adequate response but with severe hematologic toxicity [1]. One study reported acute and persistent remission in a 79‐year‐old patient with ANKL using chidamide combined with cyclophosphamide, doxorubicin, vincristine, and prednisone therapy (CHOP) [27]. The role of histone deacetylase inhibitors in combination with chemotherapy is also being explored, particularly in older patients [27]. Allogeneic HSCT remains the only curative strategy, though its feasibility is often constrained by early relapse and poor performance status [2]. Given our patient’s advanced age, dialysis dependence, and profound cytopenias, SMILE posed a substantial toxicity risk, and thus VIDL, which omits methotrexate while maintaining asparaginase‐driven cytotoxicity, was chosen [1]. Our patient initially responded to a renally adjusted VIDL regimen, with resolution of disease markers and clinical improvement. However, relapse by Day 27 precluded HSCT, underscoring the urgency of early transplant consideration.

### 3.7. Treatment and Management of HLH in ANKL Patients

The management of HLH in the setting of ANKL requires a comprehensive approach that addresses both conditions. HLH commonly is the initial presentation of ANKL, making early diagnosis crucial. Diagnostic criteria include persistent fever, cytopenias, hyperferritinemia, elevated soluble CD25 (> 2400 pg/mL), and hypertriglyceridemia [9, 21]. The positive predictive value of both high sCD25 and ferritin levels for HLH can reach 95.6% [9, 16]. Our patient exhibited profoundly elevated sCD25 (86,625 pg/mL) and CXCL9 (120,173 pg/mL), reflecting the severity of the hyperinflammatory response, and met clinical diagnostic criteria for HLH as seen in Table 1.

HLH‐directed therapy alone may delay critical opportunities for diagnosis and curative treatment of the underlying ANKL. The HLH‐2004 protocol is a regimen that includes a combination of etoposide, dexamethasone, and cyclosporine A, which has been used to effectively control EBV‐associated HLH in children without underlying disease in more than 90% of patients [16, 26]. For tumor‐associated HLH, studies suggest that using the HLH‐2004 treatment protocol can effectively control disease progression [16]. In cases where HLH is the initial manifestation of ANKL, intensive combination chemotherapy based on asparaginase and anthracycline may be considered for ANKL [6, 16]. Allogeneic HSCT is a treatment option for ANKL, even when it manifests as HLH, and can lead to long‐term disease‐free survival in some cases [6, 16]. One case reported survival of 19 months after transplant following chemotherapy for ANKL with initial HLH and lung involvement [16]. Early recognition of the potential for an underlying malignancy like ANKL in HLH cases is crucial for timely and appropriate intervention.

### 3.8. Prognosis

ANKL carries a grim prognosis, with a median overall survival of two to three months and < 10% surviving beyond one year [10, 11]. Early mortality is often driven by multiorgan failure, disseminated intravascular coagulation, or complications of associated HLH. Adverse prognostic factors include older age, EBV positivity, TP53 mutation, hyperferritinemia, HLH, and elevated lactate dehydrogenase—all present in our case [2, 10, 28]. While chemotherapy regimens such as SMILE or VIDL may induce transient remissions, relapse is common. Durable survival has been reported primarily in patients who undergo allogeneic HSCT during their first remission [1, 10].

### 3.9. Case Discussion

Most reported cases of ANKL involve younger adults of East Asian descent and are characterized by rapid progression and high early mortality [6, 10]. In contrast, our patient was a 71‐year‐old Caucasian male, an age and ethnic demographic rarely represented in ANKL literature. While EBV positivity is frequently observed, our case demonstrated negative EBER ISH, likely due to early administration of high‐dose corticosteroids and etoposide for HLH, which may have suppressed detectable viral transcripts. Such discrepancies are infrequently reported and complicate diagnosis [7].

Additionally, our patient presented with HLH, marked by fever, cytopenias, hyperferritinemia, elevated soluble IL‐2 receptor (sCD25), and markedly high CXCL9 levels, consistent with cytokine‐driven hyperinflammation. Despite severe multisystem organ failure requiring mechanical ventilation, vasopressors, and dialysis, he achieved transient remission following a VIDL‐based chemotherapy regimen, with subsequent hematologic and clinical recovery, an outcome rarely described in older adults with ANKL. Molecular profiling revealed a complex karyotype, TP53 and TET2 mutations, and two novel fusion transcripts (NCOR1:CHMP2B and TNKS:GAS7), which, to our knowledge, have not been previously reported in ANKL, further distinguishing this case.

### 3.10. Future Directions

Ongoing research in ANKL emphasizes molecular characterization, targeted therapeutics, and immunotherapy to improve outcomes in this highly lethal malignancy. NGS has revealed recurrent mutations in pathways involving JAK/STAT signaling, TP53, epigenetic regulators (e.g., TET2 and CREBBP), and cell cycle control, providing a framework for precision oncology approaches [29]. These findings support the use of ex vivo drug sensitivity profiling and mutation‐guided therapies.

Emerging immune‐based strategies include PD‐1/PD‐L1 checkpoint inhibitors, which may be particularly effective in EBV‐positive cases due to the viral‐mediated PD‐L1 upregulation [30, 31]. Chimeric antigen receptor‐engineered NK (CAR‐NK) cell therapy is a promising off‐the‐shelf, low‐toxicity alternative to CAR‐T, though further investigation in ANKL‐specific models is required [29]. Additionally, agents targeting BCL2, transferrin receptor 1, Exportin 1, and histone deacetylases (e.g., chidamide) are under investigation, with some demonstrating synergy with standard chemotherapy [29, 31].

To facilitate early diagnosis, development of standardized immunophenotypic and molecular criteria is essential, especially in EBV‐negative or HLH‐presenting cases. Prospective studies are critical to validate the correlation with novel biomarkers (e.g., CXCL9 and sCD25) and to optimize the timing of HSCT. Ultimately, collaborative multicenter studies, global data sharing, and patient registries will be critical to advancing our understanding and management of this rare malignancy.

## 4. Conclusion

ANKL remains a challenging hematological malignancy due to its rarity, aggressive nature, and variable clinical presentations. Our patient met key diagnostic criteria per the WHO 5th edition classification, including systemic illness, leukemic blood picture, and NK‐cell phenotype (CD2+, CD56+, and CD3–). While EBER ISH was negative, we postulate that aggressive treatment for the patient’s HLH resulted in this finding. The identification of TP53 and TET2 mutations further supports the diagnosis and reflects the aggressive nature of the disease.

In our patient, initial response to VIDL chemotherapy was encouraging, but relapse occurred within weeks, consistent with the dismal prognosis of ANKL. While traditional chemotherapy has limited success, L‐asparaginase‐based regimens followed by HSCT offer the most promising approach [1]. Although HSCT may offer a curative option in select patients, comorbidities, age, and rapid deterioration often limit feasibility. This case emphasizes the need for rapid diagnostic evaluation using flow cytometry, cytogenetics, and NGS in suspected ANKL cases. Continued research into the molecular pathogenesis of ANKL is essential for developing more effective and targeted therapies for improved outcomes.

## Funding

The authors did not receive any funding, grants, or other support to assist with the preparation of this manuscript.

## Ethics Statement

Informed consent to publish this case report was obtained from the patient’s medical power of attorney. The study was granted an exemption by the appropriate institutional and/or national research ethics committee, the Eastern Virginia Medial School Institutional Review Board, and is certified that the study was performed in accordance with the ethical standards as laid down in the 1964 Declaration of Helsinki and its later amendments or comparable ethical standards.

## Conflicts of Interest

The authors declare no conflicts of interest.

## Supporting Information

Please see the supporting document for a detailed report of the patient’s laboratory workup throughout the hospitalization.

## Supporting information


**Supporting Information** Additional supporting information can be found online in the Supporting Information section.

## Data Availability

The data that support the findings of this study are available on request from the corresponding author. The data are not publicly available due to privacy or ethical restrictions.

## References

[1] Sumbly V. , Vest M. , and Landry I. , Aggressive Natural Killer Cell Leukemia: A Brief Overview of Its Genomic Landscape, Histological Features, and Current Management, Cureus. (2022) 10.7759/cureus.22537. PMC8956279 35345687

[2] Peng X.-H. , Zhang L.-S. , Li L.-J. , Guo X.-J. , and Liu Y. , Aggressive Natural Killer Cell Leukemia With Skin Manifestation Associated With Hemophagocytic Lymphohistiocytosis: A Case Report, World Journal of Clinical Cases. (2021) 9, no. 34, 10708–10714, 10.12998/wjcc.v9.i34.10708. 35005005 PMC8686140

[3] Alaggio R. , Amador C. , Anagnostopoulos I. et al., The 5th Edition of the World Health Organization Classification of Haematolymphoid Tumours: Lymphoid Neoplasms, Leukemia. (2022) 36, no. 7, 1720–1748, 10.1038/s41375-022-01620-2. 35732829 PMC9214472

[4] Swerdlow S. H. , Campo E. , Pileri S. A. et al., The 2016 Revision of the World Health Organization Classification of Lymphoid Neoplasms, Blood. (2016) 127, no. 20, 2375–2390, 10.1182/blood-2016-01-643569, 2-s2.0-84971566127. 26980727 PMC4874220

[5] Lima M. , Aggressive Mature Natural Killer Cell Neoplasms: From Epidemiology to Diagnosis, Orphanet Journal of Rare Diseases. (2013) 8, no. 1, 10.1186/1750-1172-8-95. PMC3770456 23816348

[6] Amisha F. , Malik P. , Konda M. , Kakadia S. , Fugere T. , and Mukherjee A. , Epidemiology of Aggressive NK-Cell Leukemia in the United States: A SEER Population-Based Study, Blood. (2021) 138, no. S1, 10.1182/blood-2021-154381.

[7] Nicolae A. , Ganapathi K. A. , Pham T. H.-T. et al., EBV-Negative Aggressive NK-Cell Leukemia/Lymphoma, American Journal of Surgical Pathology. (2017) 41, no. 1, 67–74, 10.1097/PAS.0000000000000735, 2-s2.0-84987870736. 27631517 PMC5159195

[8] Dressler S. and Mordstein V. , Aggressive Natural Killer Cell Leukemia Associated With Hemophagocytic Lymphohistiocytosis, Blood. (2016) 128, no. 19, 10.1182/BLOOD-2016-08-732370, 2-s2.0-84994480364. 28829754

[9] Konkol S. , Killeen R. B. , and Rai M. , Hemophagocytic Lymphohistiocytosis, 2025, StatPearls Publishing. 32491708

[10] Hussein S. El , Medeiros L. , and Khoury J. , Aggressive NK Cell Leukemia: Current State of the Art, Cancers (Basel). (2020) 12, no. 10, 10.3390/cancers12102900. PMC7600035 33050313

[11] Nazarullah A. , Don M. , Linhares Y. , Alkan S. , and Huang Q. , Aggressive NK-Cell Leukemia: A Rare Entity With Diagnostic and Therapeutic Challenge, Human Pathology: Case Reports. (2016) 4, 32–37, 10.1016/j.ehpc.2015.08.001, 2-s2.0-84939817197.

[12] Küçük C. , Jiang B. , Hu X. et al., Activating Mutations of STAT5B and STAT3 in Lymphomas Derived From γδ-T or NK Cells, Nature Communications. (2015) 6, no. 1, 10.1038/ncomms7025, 2-s2.0-84923076524. PMC7743911 25586472

[13] Guerreiro M. , Príncipe F. , Teles M. J. et al., CD56-Negative Aggressive NK Cell Leukemia Relapsing as Multiple Cranial Nerve Palsies: Case Report and Literature Review, Case Reports in Hematology. (2017) 2017, 3724017–3724019, 10.1155/2017/3724017. 29163992 PMC5661071

[14] Haverkos B. M. , Pan Z. , Gru A. A. et al., Extranodal NK/T Cell Lymphoma, Nasal Type (ENKTL-NT): An Update on Epidemiology, Clinical Presentation, and Natural History in North American and European Cases, Current Hematologic Malignancy Reports. (2016) 11, no. 6, 514–527, 10.1007/s11899-016-0355-9, 2-s2.0-84992206507. 27778143 PMC5199232

[15] Fujimoto A. and Suzuki R. , Progress in the Treatment of NK-Cell Lymphoma/Leukemia, Journal of Cancer Metastasis and Treatment. (2021) 10.20517/2394-4722.2021.157.

[16] Yang R. , Ai Y. , Liu C. , and Lu X. , Aggressive Natural Killer Cell Leukemia in an Adolescent Patient: A Case Report and Literature Review, Frontiers in Pediatrics. (2022) 10, 10.3389/fped.2022.829927. PMC9168658 35676895

[17] de Leval L. , Feldman A. L. , Pileri S. , Nakamura S. , and Gaulard P. , Extranodal T- and NK-Cell Lymphomas, Virchows Archiv. (2023) 482, no. 1, 245–264, 10.1007/s00428-022-03434-0. 36336765 PMC9852223

[18] Suzuki R. , Suzumiya J. , Nakamura S. et al., Aggressive Natural Killer-Cell Leukemia Revisited: Large Granular Lymphocyte Leukemia of Cytotoxic NK Cells, Leukemia. (2004) 18, no. 4, 763–770, 10.1038/SJ.LEU.2403262, 2-s2.0-16544383785. 14961041

[19] Nakashima Y. , Tagawa H. , Suzuki R. et al., Genome-Wide Array-Based Comparative Genomic Hybridization of Natural Killer Cell Lymphoma/Leukemia: Different Genomic Alteration Patterns of Aggressive NK-Cell Leukemia and Extranodal Nk/T-Cell Lymphoma, Nasal Type, Genes Chromosomes & Cancer. (2005) 44, no. 3, 247–255, 10.1002/GCC.20245, 2-s2.0-25844434275. 16049916

[20] Ni Y. , Li L. , Wang Y. , and Sun L. , Clinicopathological Features and Treatment of Aggressive Natural Killer Cell Leukemia: Case Series and Literature Review, Turkish Journal of Pediatrics. (2024) 66, no. 4, 481–489, 10.24953/turkjpediatr.2024.5072. 39387420

[21] El-Mallawany N. K. , Curry C. V. , and Allen C. E. , Haemophagocytic Lymphohistiocytosis and Epstein-Barr Virus: A Complex Relationship With Diverse Origins, Expression and Outcomes, British Journal of Haematology. (2022) 196, no. 1, 31–44, 10.1111/BJH.17638. 34169507

[22] Kameda K. , Yanagiya R. , Miyatake Y. et al., Hepatic Niche Leads to Aggressive Natural Killer Cell Leukemia Proliferation Through Transferrin-Transferrin Receptor 1 Axis, Blood Journal. (2023) 10.1182/blood.2022018597. 37146246

[23] Zhang Y. , Lee D. , Gesiotto Q. , and Sokol L. , Aggressive Natural Killer Cell Leukemia: Diagnosis, Treatment Recommendations, and Emerging Therapies, Expert Review of Hematology. (2021) 14, no. 8, 731–740, 10.1080/17474086.2021.1955345. 34263714

[24] Kimura H. , Karube K. , Ito Y. et al., Rare Occurrence of JAK3 Mutations in Natural Killer Cell Neoplasms in Japan, Leukemia and Lymphoma. (2014) 55, no. 4, 962–963, 10.3109/10428194.2013.819577, 2-s2.0-84903815486. 23808814

[25] Dufva O. , Kankainen M. , Kelkka T. et al., Aggressive Natural Killer-Cell Leukemia Mutational Landscape and Drug Profiling Highlight JAK-STAT Signaling as Therapeutic Target, Nature Communications. (2018) 9, no. 1, 10.1038/s41467-018-03987-2, 2-s2.0-85045889211. PMC5908809 29674644

[26] Hue S. S.-S. , Oon M. L. , Wang S. , Tan S.-Y. , and Ng S.-B. , Epstein–Barr Virus-Associated T- and NK-cell Lymphoproliferative Diseases: An Update and Diagnostic Approach, Pathology. (2020) 52, no. 1, 111–127, 10.1016/j.pathol.2019.09.011. 31767131

[27] Lin Q. , Pei R. , and Lu Y. , Acute and Persistent Remission of Aggressive Natural Killer Cell Leukemia in an Older Patient Induced by Chidamide Combined With Cyclophosphamide, Vindesine, Prednisone, and Etoposide Therapy, Turkish Journal of Hematology. (2023) 40, no. 3, 225–226, 10.4274/tjh.galenos.2023.2023.0227. 37464744 PMC10476257

[28] Al-Samkari H. and Berliner N. , Hemophagocytic Lymphohistiocytosis, Annual Review of Pathology: Mechanisms of Disease. (2018) 13, no. 1, 27–49, 10.1146/annurev-pathol-020117-043625, 2-s2.0-85041748803. 28934563

[29] Liu E. , Marin D. , Banerjee P. et al., Use of CAR-Transduced Natural Killer Cells in CD19-Positive Lymphoid Tumors, New England Journal of Medicine. (2020) 382, no. 6, 545–553, 10.1056/NEJMoa1910607. 32023374 PMC7101242

[30] Yamaguchi M. , Suzuki R. , and Oguchi M. , Advances in the Treatment of Extranodal NK/T-Cell Lymphoma, Nasal Type, Blood. (2018) 131, no. 23, 2528–2540, 10.1182/blood-2017-12-791418, 2-s2.0-85048301681. 29602763

[31] Aguilar E. G. and Murphy W. J. , Obesity Induced T Cell Dysfunction and Implications for Cancer Immunotherapy, Current Opinion in Immunology. (2018) 51, 181–186, 10.1016/j.coi.2018.03.012, 2-s2.0-85045087120. 29655021 PMC6338436

