# Supplementary material for: Aggressive Natural Killer Cell Leukemia: A Rare and Rapidly Progressive Hematologic Malignancy—Case Report and Literature Review
Source: Case Rep Hematol. 2026 Apr 6;2026:7796972. doi: 10.1155/crh/7796972 (PMC13051856; doi:10.1155/crh/7796972)
Supplement: Supplementary file 1 — Supporting Information Additional supporting information can be found online in the Supporting Information section. [file CRH-2026-7796972-s001.docx]

**Supplementary Material**

*Patient’s laboratory work up throughout hospitalization*

Title: Aggressive Natural Killer Cell Leukemia: A Rare and Rapidly Progressive Hematologic Malignancy — Case Report and Literature Review

Journal Name: Annals of Hematology

Author Names: Jennifer Priessnitz MD^1^, Ali Hariri^2^, Yuliya Levkiavska MD^3^, Kyle E. Bonner MD^4^, Stephen I. Fisher MD^5^, Joshua M. Sill MD^3^

^1^Department of Internal Medicine, Eastern Virginia Medical School at Old Dominion University, Norfolk, VA, USA

^2^Department of Medicine, Eastern Virginia Medical School at Old Dominion University, Norfolk, VA, USA

^3^Division of Pulmonary Critical Care Medicine, Department of Internal medicine, Eastern Virginia Medical School at Old Dominion University, Norfolk, VA, USA

^4^Department of Diagnostic Radiology, Eastern Virginia Medical School at Old Dominion University, Norfolk, VA, USA

^5^Division of Hematopathology, Department of Pathology, Pathology Sciences Medical Group, Sentara Norfolk General Hospital, Norfolk, VA USA

Jennifer Priessnitz: [priessjk@odu.edu](mailto:priessjk@odu.edu)

Ali Hariri: 19alihariri@gmail.com

Yuliya Levkiavska: levkiay@odu.edu

Kyle E. Bonner: bonnerke@odu.edu

Stephen I. Fisher: sifisher@sentara.com

Joshua M. Sill: silljm@odu.edu

**Laboratory Test Reference Limits and Qualitative Results**

| **Category** | **Test** | **Lower Limit** | **Upper Limit** | **Units / Qualitative** |
| --- | --- | --- | --- | --- |
| **Hematology** | WBC | 3 | 16 | K/uL |
|  | RBC | 2 | 3.48 | M/uL |
|  | Hemoglobin (Hgb) | 6.2 | 10.4 | g/dL |
|  | Hematocrit (Hct) | 19.6 | 21.1 | % |
|  | MCV | 85 | 97 | fL |
|  | MCH | 28 | 31 | pg |
|  | MCHC | 31 | 36 | g/dL |
|  | RDW | 15.8 | 19.7 | % |
|  | Platelets (Plt) | 24 | 106 | K/uL |
|  | MPV | 10.3 | 14 | fL |
|  | Immature Platelet Fraction | 4 | 18.6 | % |
|  | Segmented Neutrophils | 62 | - | % |
|  | Lymphocytes | 12 | - | % |
|  | Monocytes | 25 | - | % |
|  | Eosinophils | 0 | - | % |
|  | Basophils | 0 | - | % |
| **Coagulation** | D-dimer | 2.32 | 5.48 | mg/L FEU |
|  | AdamTS13 | 0.37 | - | IU/mL |
| **General Chemistry** | BUN | 24 | 126 | mg/dL |
|  | Creatinine (Cr) | - | 3.7 | mg/dL |
|  | eGFR | - | 16.9 | mL/min |
|  | Sodium (Na⁺) | 124 | - | mmol/L |
|  | Potassium (K⁺) | 3.5 | 5.1 | mmol/L |
|  | Chloride (Cl⁻) | 100 | 113 | mmol/L |
|  | CO₂ | 14 | 26 | mmol/L |
|  | Anion Gap | 11 | 19 | mmol/L |
|  | Calcium | 7.0 | 9.0 | mg/dL |
|  | Calcium (Ionized) | 3.8 | 4.2 | mg/dL |
|  | Magnesium | 1.6 | 3.6 | mg/dL |
|  | Phosphate | 2.9 | 7.9 | mg/dL |
|  | Uric Acid | 2.7 | 10.6 | mg/dL |
|  | AST | 43 | 224 | U/L |
|  | ALT | 16 | 60 | U/L |
|  | LDH | 551 | 881 | U/L |
|  | Alk Phosphatase | 191 | 539 | U/L |
|  | Total Bilirubin | 0.4 | 1.9 | mg/dL |
|  | Direct Bilirubin | - | 0.7 | mg/dL |
|  | Total Protein | 4.6 | 6.1 | g/dL |
|  | Albumin | 2.4 | 3.4 | g/dL |
|  | Globulin | 1.9 | 3.0 | g/dL |
|  | A/G Ratio | 1 | 1.6 |  |
|  | GGT | - | 146 | U/L |
|  | Lipase | - | 37 | U/L |
|  | Iron | - | 26 | mcg/dL |
|  | UIBC | - | 185 | mcg/dL |
|  | TIBC | - | 211 | mcg/dL |
|  | Iron Saturation | 12 | - | % |
|  | Ferritin | 2000 | 3339 | ng/mL |
|  | Ammonia | - | 35 | mcg/dL |
|  | Triglycerides | 268 | 615 | mg/dL |
| **Urinalysis** | Glucose |  | Negative |  |
|  | Ketones | Trace |  |  |
|  | Bilirubin |  | Negative |  |
|  | Blood |  | Negative |  |
|  | Nitrite |  | Negative |  |
|  | Leukocyte Esterase |  | Negative |  |
|  | Ictotest |  | Negative |  |
|  | Urobilinogen | 0.2 | 1.0 | mg/dL |
| **Infectious Disease** | HIV1/0/2 Ab/Ag |  | Non-reactive |  |
|  | Hepatitis A IgM Ab |  | Non-reactive |  |
|  | Hepatitis B core IgM Ab |  | Non-reactive |  |
|  | HBsAg |  | Non-reactive |  |
|  | Hepatitis C Ab |  | Non-reactive |  |
|  | C. Diff PCR |  | Negative |  |
|  | Cryptococcal Antigen |  | Negative |  |
|  | EBV PCR |  | 780 | copies/mL |
|  | VDRL (CSF) |  | Non-reactive |  |
|  | Enterovirus PCR |  | Not Detected |  |
|  | Lyme PCR |  | Not Detected |  |
|  | Bartonella henselae IgG |  | Negative |  |
|  | Bartonella henselae DNA |  | Not Detected |  |
|  | CSF West Nile IgG | <1.30 |  | Index |
|  | CSF West Nile IgM | <0.90 |  | Index |
| **Tumor Markers** | AFP Tumor Marker |  | <1.8 | ng/mL |
|  | CA 19-9 |  | 20.4 | U/mL |
|  | CEA |  | 1.2 | ng/mL |
|  | Chromogranin A |  | 419 | ng/mL |
|  | Neuron Specific Enolase |  | 140 | ng/mL |

**Viral PCR and Immunological testing:**

Soluble CD25: 86,625 pg/mL

9: 120,173 pg/mL

Neuronal Nuclear Ab, Type 1 (Hu): Negative

Neuronal Nuclear Ab, Type 2 (Ri): Negative

Neuronal Nuclear Ab, Type 3: Negative

Purkinje Cell Cytop. Ab, Type 1 (Yo): Negative

Purkinje Cell Cytop. Ab, Type 2: Negative

Purkinje Cell Cytop. Ab, Type Tr: Negative

Glial Nuclear Ab, Type 1: Negative

Calcium Channel Ab P/Q-Type: <30 pmol/L

Neuronal (V-G) K+Channel Ab, S: <80 pmol/L

AChR Ganglionic Neuronal Ab, S: <55 pmol/L

Calcium Channel, Ab N-Type:  <54 pmol/L

Striational Muscle Ab: Negative

Acetylcholine Receptor Binding Ab: <0.30 nmol/L

Amphiphysin Ab, S: Negative

CRMP-5 IgG, S: Negative

Cryptococcal Antigen: Negative

Cysticercosis Ab, IgG ELISA: <0.75

Enterovirus Real time PCR: Not Detected

Lyme Disease DNA PCR: Not Detected

VDRL CSF: Non-Reactive

Neuron Specific Enolase Serum: 140 ng/mL

GBM Antibody: <1.0 AI

Myeloperoxidase Antibody: <1.0 AI

Neutrophil Cytoplasmic Antibody: Negative

Proteinase-3 Antibody: <1.0 AI

Bartonella henselae IgG: Negative

Bartonella quintana IgG: Negative

Bartonella henselae IgM: Negative

Bartonella quintana IgM: Negative

Bartonella henselae DNA: Not Detected

Bartonella quintana DNA: Not Detected

CSF West Nile IgG: <1.30 Index

CSF West Nile IgM: <0.90 Index

HIV1/0/2 Ab/Ag: Non Reactive

ANA Antibodies: Negative

Neutrophil Cytoplasmic Antibody: Negative

Herpes 1 PCR CSF: Negative

EBV-PCR QN: 780 copies/mL

EBV-VCA Abs: IgM <0.2 AI

TCRG Gene: Negative

TCRB Gene: Negative
